# Watchful waiting for subthreshold depression and anxiety in visually impaired older adults

**DOI:** 10.1007/s11136-015-1032-5

**Published:** 2015-06-18

**Authors:** Hilde P. A. van der Aa, Esther Krijnen-de Bruin, Ger H. M. B. van Rens, Jos W. R. Twisk, Ruth M. A. van Nispen

**Affiliations:** Department of Ophthalmology, VU University Medical Centre, De Boelelaan 1117, 1081 HV Amsterdam, The Netherlands; EMGO+Institute for Health and Care Research (EMGO+), VU University Medical Centre, Van der Boechorststraat 7, 1081 BT Amsterdam, The Netherlands; Department of Ophthalmology, Elkerliek Hospital, Wesselmanlaan 25, 5707 HA Helmond, The Netherlands; Department of Epidemiology and Biostatistics, VU University Medical Centre, De Boelelaan 1117, 1081 HV Amsterdam, The Netherlands

**Keywords:** Watchful waiting, Low vision, Depression, Anxiety

## Abstract

**Purpose:**

Immediate treatment of depression and anxiety may not always be necessary in resilient patients. This study aimed to determine remission rates of subthreshold depression and anxiety, incidence rates of major depressive and anxiety disorders, and predictors of these remission and incidence rates in visually impaired older adults after a three-month ‘watchful waiting’ period.

**Methods:**

A pretest–posttest study in 265 visually impaired older adults (mean age 74 years), from outpatient low-vision rehabilitation services, with subthreshold depression and/or anxiety was performed as part of a randomised controlled trial on the cost-effectiveness of a stepped-care intervention. An ordinal logistic regression analysis was conducted. Main outcome measures were: (1) subthreshold depression and anxiety measured with the Centre for Epidemiologic Studies Depression Scale (CES-D) and the anxiety subscale of the Hospital Anxiety and Depression Scale (HADS-A), and (2) depressive and anxiety disorders measured with the Mini International Neuropsychiatric Interview.

**Results:**

After a three-month watchful waiting period, depression and anxiety decreased significantly by 3.8 (CES-D) and 1.4 points (HADS-A) (*p* < 0.001). Of all participants, 34 % recovered from subthreshold depression and/or anxiety and 18 % developed a depressive and/or anxiety disorder. Female gender [odds ratio (OR) 0.49, 95 % confidence interval (CI) 0.28–0.86], more problems with adjustment to vision loss at baseline (OR 1.02, 95 % CI 1.00–1.03), more symptoms of depression and anxiety at baseline (OR 1.06, 95 % CI 1.02–1.10), and a history of major depressive, dysthymic, and/or panic disorder (OR 2.28, 95 % CI 1.28–4.07) were associated with lower odds of remitting from subthreshold depression and/or anxiety and higher odds of developing a disorder after watchful waiting.

**Conclusions:**

Watchful waiting can be an appropriate step in managing depression and anxiety in visually impaired older adults. However, female gender, problems with adjustment to vision loss, higher depression and anxiety symptoms, and a history of a depressive or anxiety disorder confer a disadvantage. Screening tools may be used to identify patients with these characteristics, who may benefit more from higher intensity treatment or a shorter period of watchful waiting.

## Introduction

Current European and American guidelines in mental health care recommend a period of ‘active monitoring’ or ‘watchful waiting’ as a first step to deal with mild symptoms of depression and anxiety [1–3]. Watchful waiting involves an active decision of a clinician and patient not to immediately treat the condition but, instead, to intermittently reassess its status after a certain period of time [4–6]. Watchful waiting may prevent overtreatment and reduce healthcare costs [4–6]. It is often the first step in a stepped-care approach, in which subsequent treatment components are offered by order of intensity [7–10]. It may be an adequate approach for subthreshold depression and anxiety (indicating clinically significant symptoms, but no actual disorder) since the majority of patients in the general population remits from these conditions without offering active treatment [5, 11, 12].

However, it is unclear whether watchful waiting would also suit the vulnerable population of visually impaired older adults. Vision loss affects about 285 million people globally, of whom 65 % are aged 50 years or older [13]. It is one of the leading causes of age-related disability and can lead to reduced quality of life and higher levels of depression and anxiety [14–19]. About one-third of visually impaired older adults experience subthreshold depression and/or anxiety; 5–7 % are diagnosed with a major depressive disorder and 7 % with an anxiety disorder [14–19]. These percentages are substantially higher than the prevalence in normally sighted peers [14, 20–22]. Both disorders can have a detrimental impact on visually impaired older adults, leading to decreased quality of life, decline in health status, increased vision-specific disability, and even mortality [16, 23–25]. Because of the high comorbidity and symptom overlap of depression and anxiety in visually impaired older adults, a focus on a combination of these conditions is sensible [14].

In addition, it would be interesting to find out which factors predict change in depression and anxiety in visually impaired older adults after watchful waiting to indicate for which patients this step would be (in)appropriate. Previous studies indicate that multiple factors may influence depression and anxiety in visually impaired older adults, e.g. gender, age [19], perceived vision-specific disability [26], adaptation to vision loss [27, 28], perceived physical condition [17, 19, 26, 29], somatic and psychiatric comorbidities [19, 29], and a history of major depressive disorder [29]. However, these studies were all cross-sectional.

The objectives of the present study were to examine: (1) the remission rate of subthreshold depression and/or anxiety, (2) the incidence of major depressive, dysthymic, and anxiety disorders, and (3) covariates that predicted remission and incidence rates in visually impaired older adults (aged > 50 years) with subthreshold depression and/or anxiety after a three-month period of watchful waiting. The outcomes may offer important information for researchers, clinicians, and policymakers in the field of low vision to deal with depression and anxiety.

We hypothesised that demographic variables (gender, age, education, living situation, income), vision-specific variables (visual acuity, cause of vision loss, time of onset), comorbidity, vision-related quality of life, adaptation to vision loss, symptoms of depression and anxiety, and history of depressive and anxiety disorders, as measured at baseline, would be associated with change in the outcome after watchful waiting. In addition, we took mental health services that were used during watchful waiting into account.

## Methods

### Design

A pretest–posttest study was conducted among 265 visually impaired older adults (≥50 years) from outpatient low-vision rehabilitation centres, with subthreshold depression and/or anxiety. Data were collected from September 2012 to January 2014 as part of a randomised controlled trial (RCT) to investigate the cost-effectiveness of a stepped-care programme to prevent depressive and anxiety disorders in visually impaired older adults (trial registration: http://www.trialregister.nl, identifier: NTR3296) [30]. Unmasked participants were randomised to either the intervention group (receiving the stepped-care programme in addition to usual care) or the control group (receiving usual care only). Data were collected at baseline and after 3 months by means of telephone interviews performed by masked research assistants who were trained according to a pre-specified protocol.

### Participants

A total of 3000 patients (aged ≥ 50 years) who were registered at an outpatient low-vision rehabilitation centre in the Netherlands or Flanders (the Dutch-speaking part of Belgium) were invited to participate. Of these, 914 provided written consent (response rate 30.5 %). In these patients, eligibility was determined based on: (a) having subthreshold depression and/or anxiety (a score of ≥16 on the Centre for Epidemiologic Studies Depression Scale (CES-D) [31–33] and/or a score of ≥8 on the anxiety subscale of the Hospital Anxiety and Depression Scale (HADS-A) [34, 35]), (b) not meeting the full diagnostic criteria for a depressive and/or anxiety disorder (based on the Mini International Neuropsychiatric Interview (MINI) [36, 37]), (c) speaking the Dutch language adequately, and (d) not being severely cognitively impaired (based on the six-item screener, a short version of the Mini-Mental State Examination (MMSE) [38]).

### Intervention

The first step of the stepped-care programme was a three-month period of watchful waiting. During this step, the executive researcher contacted patients by telephone at baseline and after 3 months (±15 min each), and patient could contact the executive researcher during this period if necessary. A three-month period was chosen based on a previously found effective stepped-care programme in community-dwelling elderly [10] and on the premise that all participants received usual care of low-vision rehabilitation centres, which could already positively influence the reduction in depressive and anxiety symptoms [39, 40]. Despite randomisation, both the intervention and control group received watchful waiting since no actual treatment was offered. Therefore, the analyses in the present study were performed on the total group of participants. Nevertheless, randomisation was analysed as a covariate, as the intervention group was informed about receiving subsequent treatment after watchful waiting, which may have influenced the outcome. Usual care included outpatient low-vision rehabilitation care and/or care that was provided by other healthcare providers. The use of mental health services during watchful waiting was analysed as a covariate, because this may have influenced the outcome. Additional details on this RCT and the stepped-care programme are described elsewhere [30].

### Outcome measures

#### Subthreshold depression and anxiety

The CES-D and HADS-A were used at baseline and after 3 months to determine subthreshold depression and/or anxiety. The CES-D is a 20-item questionnaire with total scores ranging from 0 to 60 and a cut-off score for subthreshold depression and/or anxiety of ≥16 [31]. The CES-D is considered a valid and reliable instrument to measure late-life depression and anxiety [32]. However, because the criterion validity of the CES-D was considerably better for depression than for anxiety [33], the HADS-A was used to measure subthreshold anxiety. The HADS-A is a 7-item subscale which specifically targets anxiety, with scores ranging from 0 to 21 and a cut-off score for subthreshold anxiety of ≥8 [34]. The reliability of the HADS-A is reported to be good to very good [35].

#### Major depressive and anxiety disorders

The MINI was used in all participants to determine the incidence of major depressive, dysthymic, and/or anxiety disorders (panic disorder, agoraphobia, social phobia, and/or general anxiety disorder) at baseline and after 3 months and to determine history of major depressive, dysthymic, and panic disorder at baseline. This brief diagnostic interview is considered a valid and reliable tool to define mental disorders according to the DSM-IV based on telephone interviews in Dutch clinical practice [36, 37].

#### Vision-related quality of life

The Low Vision Quality of Life Questionnaire (LVQOL) was used at baseline to measure vision-related quality of life, with scores ranging from 0 to 100 indicating low to high quality of life [41, 42]. The LVQOL showed internal reliability and validity and consists of four subscales: basic aspects, adjustment, reading and fine work, and mobility [42, 43]. Adaptation to vision loss was measured at baseline using the 12-item Adaptation to Vision Loss Scale (AVL-12). The AVL-12 is a short, efficient measure that shows strong psychometric properties [44].

#### Health services utilisation

Utilisation of mental health services was assessed after 3 months of watchful waiting based on the Dutch version of the Perceived Need for Care Questionnaire (PNCQ) from the Netherlands Study of Depression and Anxiety (NESDA) [45]. This is considered a reliable and valid instrument to distinguish descriptive information of the use of mental health services [46]. Six types of services were distinguished: (1) information about mental illnesses and treatment possibilities; (2) practical support, e.g. vision-specific tools or domestic help; (3) skills training; (4) counselling/therapy; (5) medication; and (6) referral to a mental health services specialist.

#### Visual acuity

Decimal visual acuity (based on the internationally used Snellen chart) was retrieved from the patient files at low-vision rehabilitation centres at baseline. Missing values (*n* = 22) were supplemented with answers that participants provided themselves based on recent ophthalmic diagnostics. These values were converted into logMAR values (−log_10_ visual acuity) in the best eye to enable meaningful computations. A logMAR visual acuity of 0.00–0.29 indicated normal vision, 0.30–0.51 indicated mild vision loss, and 0.52–2.00 indicated low vision or blindness.

#### Comorbidity

Patients were asked about comorbidity at baseline based on eight large condition groups: asthma or chronic obstructive pulmonary disease; osteoarthritis and rheumatoid arthritis; peripheral arterial disease; diabetes mellitus; cardiac disease; cerebrovascular accident or stroke; cancer; and other chronic somatic or psychiatric conditions.

### Statistical analysis

Paired sample *t* tests were performed to compare depression and anxiety scores at baseline and after watchful waiting. In order to measure predictors of change in ‘severity of depression and anxiety’, first the outcome variable was categorised into three groups: (1) no symptoms of depression and/or anxiety (CES-D score of <16 and HADS-A score of <8), (2) subthreshold depression and/or anxiety (CES-D score of ≥16 and/or HADS-A score of ≥8), and (3) major depressive, dysthymic, and/or anxiety disorder (measured with the MINI). Therefore, patients with a diagnosis of a disorder (based on the MINI) were categorised into the last group, despite their scores on the CES-D and HADS-A. This categorisation is also used in the RCT to investigate the cost-effectiveness of the total stepped-care programme to determine whether patients should move on to a higher intensity treatment component [30].

Second, a stepwise ordinal logistic regression analysis was performed (*p* ≤ 0.10) with the following covariates measured at baseline: gender, age, education, living situation, income, visual acuity, cause of vision loss, time of onset of the visual impairment, comorbidity, vision-related quality of life, adaptation to vision loss, baseline CES-D scores, baseline HADS-A scores, history of major depressive, dysthymic, and/or panic disorder, and randomisation status. Mental health services utilisation measured after 3 months was also taken into account. In addition, we checked for interaction effects of age, education, and living situation. Education was recoded into the number of years having received education. Living situation (dependent or independent), cause of vision loss (macular degeneration or other), comorbidity (having a comorbid disorder or not), and mental health services utilisation (having received some form of mental health services or not) were dichotomised. Proportionality of odds ratios (ORs) were tested with the logit link function, multicollinearity was tested with a variance inflation factor (VIF) of <3, and linearity was tested by means of dummy variables. Data analysis was performed using SPSS 20.

## Results

### Demographic and clinical characteristics

No significant difference was found in gender between responders and non-responders; however, responders were significantly younger than non-responders (mean difference = 4.6 years, *p* < 0.001). Loss to follow-up after 3 months of watchful waiting was 9.3 %. No significant difference was found between participants who dropped out and those who did not drop out in any of the relevant outcome measures. The most common reasons for participants to drop out of the study were: (1) mortality and (2) it was too great a burden to continue. Table 1 shows that the average age of the study population at baseline was 74 years; the majority of participants was female (69.8 %) and lived independently (90.2 %). Almost half of the participants had macular degeneration, and the mean time of onset was 15.2 years ago. About 21 % had a history of major depressive disorder, about 2 % had a history of dysthymic disorder, and about 6 % had a history of panic disorder. During watchful waiting, more than half of the study population received some form of mental health services. Practical support (35.0 %), information about mental illnesses and treatment possibilities (18.3 %), counselling/therapy (17.1 %), and medication (17.1 %) were received most often. These services were mostly provided by social workers and psychologists.

**Table 1:** Patient characteristics at baseline (*N* = 265) and after watchful waiting (*N* = 246)

	Total
Measured at baseline (*N* = 265)	
Gender (female) [*N* (%)]	185 (69.8 %)
Age (years), range [50–98] (mean (SD), median)	73.7 (12.3), 75.0
Education (years), range [0–16] (mean (SD), median)^a^	9.8 (3.6), 10.0
Living situation (independent) [*N* (%)]	239 (90.2 %)
Income [*N* (%)]^b^	
Usually enough money	123 (46.4 %)
Just enough money	112 (42.3 %)
Not enough money	25 (9.4 %)
LogMAR visual acuity [*N* (%)]^c^	
Normal visual acuity	24 (9.1 %)
Mild vision loss	47 (17.7 %)
Low vision or blindness	172 (64.9 %)
Cause of vision loss [*N* (%)]^d^	
Macular degeneration	122 (46.0 %)
Glaucoma	45 (17.0 %)
Cataract	45 (17.0 %)
Cerebral haemorrhage	15 (5.7 %)
Diabetic retinopathy	9 (3.4 %)
Other	105 (39.6 %)
Time of onset of the visual impairment (years), range [0–79] (mean (SD), median)^e^	15.2 (18.9), 8.0
Having one or more comorbid disorder [*N* (%)]	154 (62.6 %)
Adaptation to vision loss, range [16–48] (mean (SD))^f^	31.5 (6.1)
Vision-specific quality of life, range [0–180] [mean (SD)]	
Basic aspects^g^	54.8 (17.1)
Adjustment^h^	40.4 (20.0)
Reading and fine work^g^	50.6 (25.4)
Mobility^i^	51.2 (21.0)
History of major depressive disorder [*N* (%)]	55 (20.8 %)
History of dysthymic disorder [*N* (%)]	5 (1.9 %)
History of panic disorder [*N* (%)]	17 (6.4 %)
Measured after watchful waiting (*N* = 246)	
Mental health services utilisation [*N* (%)]	
Information	45 (18.3 %)
Practical support	86 (35.0 %)
Counselling/therapy	42 (17.1 %)
Medication	42 (17.1 %)
Referral to specialist	15 (6.1 %)
Skills training	9 (3.7 %)
None	118 (44.4 %)
Severity of depression and anxiety [*N* (%)]	
No symptoms	84 (34.1 %)
Subthreshold symptoms	117 (47.6 %)
Depressive/anxiety disorder	45 (18.3 %)

Range, means and standard deviations (SD) are reported for continuous variables, and the median is additionally provided when the variable has an asymmetric distribution. Missing observations (N:) ^a^21;^b^5;^c^22;^d^2;^e^4;^f^18;^g^23;^h^14; ^i^20

### Remission rates

Table 1 shows that after 3 months, 34.1 % remitted from subthreshold depression and/or anxiety, 47.6 % still experienced subthreshold depression and/or anxiety, and 18.3 % had progressed to a major depressive, dysthymic, and/or anxiety disorder. In the latter group, 11.4 % had developed an anxiety disorder, 10.2 % had developed a depressive disorder, and 3.3 % had developed both a depressive and anxiety disorder. Table 2 shows that there was a significant reduction in depressive and anxiety symptoms after 3 months of watchful waiting. The total CES-D score dropped 3.8 points (*p* < 0.001), while the total HADS-A score dropped 1.4 points (*p* < 0.001).

**Table 2:** Depression and anxiety symptoms at baseline and after watchful waiting

Patient characteristics	Baseline	After watchful waiting	*t*	*p*
CES-D [mean (SD)]	21.3 (6.4)	17.5 (8.7)	6.048	**<0.001**
HADS-A [mean (SD)]	7.1 (4.0)	5.7 (3.8)	4.900	**<0.001**

Bold is significant at *p* < 0.05

*CES*-*D* Centre for Epidemiologic Studies Depression Scale, *HADS*-*A* Hospital Anxiety and Depression Scale—Anxiety subscale

### Plausible predictors of improvement

No plausible multicollinearity was found, and the proportionality of ORs was met. Age and time of onset of the visual impairment had no linear relationship with the outcome measure. Therefore, the scales of these variables were changed into an ordinal scale with four equally large categories (quartiles). In addition, we found no interaction effects of age, education, and living situation (*p* > 0.05).

All hypothesised covariates were included in the model (Table 3; full model). After conducting the backward-stepwise procedure, gender, the baseline CES-D scores, the baseline scores on the adjustment subscale of the LVQOL, history of major depressive, dysthymic, and/or panic disorder proved to be significant predictors (*p* ≤ 0.10) of severity of depression and anxiety after 3 months (Table 3; final model). The final model explained 10.4 % (Cox and Snell R square) to 11.9 % (Nagelkerke R squared) of the variance in severity of depression and anxiety.

**Table 3:** Univariate and multivariate determinants of severity of depression and anxiety (higher category refers to greater severity) after watchful waiting based on an ordinal logistic regression analysis using a backwards stepwise procedure (*N* = 246)

Predictors	Univariate			Multivariate (full model)			Multivariate (end model)		
	OR	95 % CI	*p*	OR	95 % CI	*p*	OR	95 % CI	*p*
Gender (male vs. female)	0.64	0.39–1.08	0.092	0.69	0.33–1.45	0.327	0.49	0.28–0.86	0.013
*Age (years)* ^*a*^									
50–62	0.88	0.46–1.69	0.698	0.34	0.10–1.11	0.958			
63–75	0.96	0.50–1.85	0.900	0.62	0.22–1.71	0.357			
76–83	0.61	0.31–1.21	0.159	0.74	0.28–1.96	0.548			
Education (years)	0.98	0.91–1.05	0.495	0.94	0.85–1.04	0.231			
Living situation (independent vs. dependent)	0.63	0.25–1.60	0.336	1.58	0.25–9.81	0.626			
*Income* ^*b*^									
Usually enough money	0.50	0.21–1.16	0.108	1.03	0.29–3.65	0.958			
Just enough	0.48	0.20–1.13	0.092	0.29	0.25–2.94	0.801			
*Visual acuity* ^c^									
Normal vision	1.23	0.54–2.78	0.624	0.96	0.29–3.21	0.941			
Mild vision loss	1.08	0.58–2.03	0.805	0.78	0.33–1.85	0.573			
Cause of vision loss (macular degeneration vs. other)	1.46	0.91–2.34	0.117	1.27	0.60–2.69	0.531			
*Time of onset of the VI (years)* ^*d*^									
0–3	1.44	0.75–2.77	0.270	1.46	0.52–4.09	0.475			
4–7	1.60	0.78–3.25	0.200	1.38	0.47–4.07	0.562			
8–18	1.69	0.87–3.27	0.120	0.99	0.35–2.80	0.975			
Having one or more comorbid disorders versus none	1.28	0.78–2.08	0.326	1.54	0.78–3.06	0.215			
CES-D score	1.05	1.02–1.10	0.007	1.04	0.99–1.10	0.088	1.06	1.02 –1.10	0.006
HADS-A score	1.09	1.03–1.16	0.005	1.06	0.97–1.10	0.173			
Having a history of depressive/dysthymic/panic disorder versus no history	1.88	1.10–3.21	0.021	1.94	0.90–4.20	0.093	2.28	1.28–4.07	0.005
LVQOL: basic aspects	1.02	1.00–1.03	0.051	1.03	1.01–1.06	0.016			
LVQOL: adjustment	1.02	1.01–1.03	0.007	1.02	1.00–1.05	0.048	1.02	1.00–1.03	0.031
LVQOL: reading and fine work	1.01	0.99–1.02	0.216	0.99	0.97–1.01	0.154			
LVQOL: mobility	1.02	1.00–1.03	0.013	1.00	0.98–1.02	0.715			
Adaptation to vision loss	1.02	0.98–1.06	0.277	0.92	0.86–0.99	0.018			
Having used one or more mental healthcare services versus none	1.42	0.89–2.28	0.145	1.64	0.83–3.24	0.159			
Randomisation (control group vs. intervention group)	1.14	0.71–1.83	0.580	0.70	0.36–1.37	0.301			

Positive estimates indicate a higher category of the outcome compared to the reference

Reference group: a, age 84–98, b, not enough money; c, low vision/blindness; d, 19–79 years ago

*CES*-*D* Centre for Epidemiologic Studies Depression Scale, *HADS*-*A* Hospital Anxiety and Depression Scale—Anxiety subscale, *LVQOL* Low Vision Quality of Life Questionnaire

The model indicated that female gender [odds ratio (OR) 0.49, 95 % confidence interval (CI) 0.28–0.86], more symptoms of depression and anxiety at baseline (OR 1.06, 95 % CI 1.02–1.10), more problems with adjustment to vision loss at baseline (OR 1.02, 95 % CI 1.00–1.03), and a history of major depressive, dysthymic, and/or panic disorder (OR 2.28, 95 % CI 1.28–4.07) were associated with higher odds of developing a major depressive, dysthymic, and/or anxiety disorder and lower odds of remitting from subthreshold depression and/or anxiety after watchful waiting. Randomisation did not appear to be a predictor, confirming that it was appropriate to perform analysis on the total group of participants.

## Discussion

The present study indicates that watchful waiting can be an appropriate first step for visually impaired older adults with mild symptoms of depression and/or anxiety. During this period, one in three participants recovered from subthreshold depression and/or anxiety. Community-based surveys suggest remission rates in more than half of the cases [11, 12]. Our percentage is lower, but can be expected because our population is less resilient based on a higher age and visual impairment. Hegel et al. [5] reported substantially lower remission rates of 9–13 % in primary care patients after a follow-up period of one month. This difference may be explained by the shorter time period chosen by Hegel et al. [5] and the low percentage of eligible participants that enrolled and remained in their study throughout the watchful waiting period.

In contrast, a reasonably high percentage (18 %) of our study population developed a major depressive, dysthymic, and/or anxiety disorder during watchful waiting. Female patients with more symptoms of depression and/or anxiety, more problems with adjustment to vision loss, and a history of major depressive, dysthymic, and/or panic disorder had higher odds of developing a disorder during watchful waiting, which is in line with previous studies [27–29]. For these patients, watchful waiting may not be an appropriate step. They may benefit more from a higher intensity treatment or a shorter period of watchful waiting with more frequent monitoring. Current guidelines of the National Institute for Health and Care Excellence (NICE) in the UK recommend using a watchful waiting period of 2 weeks, which may be more appropriate [2].

Screening questionnaires may be used to identify patients that are less resilient in overcoming subthreshold depression and anxiety. A brief version of the Patient Health Questionnaire (PHQ), which can be used by non-mental health staff to screen for depression and anxiety, may be suitable for this purpose [47, 48]. When patients experience depression and/or anxiety based on this short screener, extensive questionnaires can be used to determine whether watchful waiting is appropriate, in which history of depressive and anxiety disorders should be taken into account.

### Strengths and limitations

The present study has several strengths. It is the first study to investigate watchful waiting in visually impaired older adults, and only a few other studies have investigated watchful waiting in the general population [5, 11, 12]. Therefore, the outcomes may be meaningful for both research and clinical practice. It may offer important indications for clinicians and policymakers to deal with depression and anxiety in the field of low vision. In addition, validated questionnaires were used to analyse symptoms of depression and anxiety as well as actual disorders according to the DSM-IV, enabling to examine remission rates of depression and anxiety as well as incidence rates of actual disorders.

However, this study also has some limitations. Only treatment-seeking patients (i.e. outpatient low-vision rehabilitation services), who enrolled in a randomised controlled study, were included in the present study. Only 30.5 % of the invited patients provided written informed consent, and these responders were significantly younger than non-responders. In addition, study participants may have been relatively healthier (i.e. able to take part in the interviews and not cognitively impaired) and may have had higher needs for and better access to health services. This reduces generalisability of the outcomes.

Furthermore, it should be noted that more than half of the participants received some form of mental health services during watchful waiting, as they were all in care of outpatient low-vision rehabilitation centres and were free to seek help elsewhere if they wanted to. This may have undermined the actual goal of watchful waiting, i.e. not receiving professional mental health services. However, received mental health services did not prove to be a significant predictor of the outcome measure. In addition, the final model only explained 10.4–11.9 % of the variance in severity of depression and anxiety. Further research is needed to investigate other constructs (e.g. social support, family history of depression) that may influence the outcome.

## Conclusion

Watchful waiting appears to be an effective first step in dealing with subthreshold depression and anxiety in visually impaired older adults. However, female gender, problems with adjusting to vision loss, higher depression and anxiety symptoms, and a history of major depressive, dysthymic, and/or panic disorder confer a disadvantage. Short screening tools may be used to identify patients for whom watchful waiting may be less appropriate. These patients may benefit more from a higher intensity treatment or a shorter period of watchful waiting. Since evidence for watchful waiting is limited, future studies are needed to confirm our findings and to determine the most appropriate time period for different patient groups.

## Abbreviations

RCT: Randomised controlled trial
CES-D: Centre for Epidemiologic Studies Depression Scale
HADS-A: Hospital Anxiety and Depression Scale—Anxiety subscale
MINI: Mini International Neuropsychiatric Interview
LVQOL: Low Vision Quality of Life Questionnaire
AVL: Adaptation to Vision Loss Scale
PNCQ: Perceived Need for Care Questionnaire
OR: Odds ratio
